# Supplementary material for: Structures of outer-arm dynein array on microtubule doublet reveal a motor coordination mechanism
Source: Nat Struct Mol Biol. 2021 Sep 23;28(10):799–810. doi: 10.1038/s41594-021-00656-9 (PMC8500839; doi:10.1038/s41594-021-00656-9)
Supplement: Supplementary file 1 — Supplementary Table 1 [file 41594_2021_656_MOESM1_ESM.pdf]

nature portfolio

https://doi.org/10.1038/s41594-021-00656-9

Supplementary information

Structures of outer-arm dynein array on microtubule doublet reveal a motor coordination mechanism

In the format provided by the authors and unedited

nature portfolio

https://doi.org/10.1038/s41594-021-00656-9

Supplementary information

Structures of outer-arm dynein array on microtubule doublet reveal a motor coordination mechanism

In the format provided by the
authors and unedited

Supplementary Table 1: A summary of OAD subunits.

Subunit name (this paper)	Gene Model Identifier	TGD standard name	MW (KDa)	Uniprot ID	MS (significant matches)	Orthologues in *C.reinhardtii* (Uniprot ID)
**α-HC**	TTHERM_01276420	**DYH3**	534	Q22A67	1598	**γ-HC** (Q39575)
**β-HC**	TTHERM_00499300	**DYH4**	530	I7M9J2	1635	**β-HC** (Q39565)
**γ-HC**	TTHERM_00486600	**DYH5**	475	I7M6H4	1518	**α-HC** (Q39610)
**IC2**	TTHERM_00487150	**DIC2**	78	I7M008	280	**IC1** (Q39578)
**IC3**	TTHERM_00079230	**DIC3**	77	Q23FU1	397	**IC2** (P27766)
**LC7-a**	TTHERM_00348650	N/A	15	I7MHB1	72	**LC7a** (AAD45881)
**LC7-b**	TTHERM_00030210	**LC7B**	12	Q22MV2	73	**LC7b** (EDP03034)
**LC8-1b**	TTHERM_00716250	**LC10**	12	I7MCM8	25	**LC10** (EDP00562)
**LC8-1a**	TTHERM_00449029	**DLC82**	11	A4VE64	37	**LC8** (Q39580)
**LC8-2b**	TTHERM_00023950	N/A	12	Q22R86	29	
**LC8-2a**	TTHERM_00971840	**LC8E**	11	Q24DI9	15	
**LC8-3b**	TTHERM_000442909	N/A	13	W7XJB1	36	
**LC8-3a**	TTHERM_01079060	N/A	10	Q24CE5	39	
**Tctex-a**	TTHERM_00392979	N/A	13	A4VEB3	16	**LC9** (DAAZ95589)
**Tctex-b**	N/A	N/A	14	Q1HGH8	22	
**LC3BL**	TTHERM_00149859	N/A	13	A4VD75	44	**LC3** (Q39592)
**LC1**	TTHERM_00334290	**LC1**	22	I7M1N7	88	**LC1** (AAD41040)
**LC4A**	TTHERM_01075620	**LC4A**	18	Q22C78	21	**LC4** (Q39584)
**α-tubulin**	TTHERM_00558620	**ATU1**	49	I7M9N6	7	**TUA1** (Q540H1)
**β-tubulin**	TTHERM_00836580	**BTU2**	49	Q24B92	11	**TUB1** (A8IXZ0)
