# Supplementary material for: Structures of outer-arm dynein array on microtubule doublet reveal a motor coordination mechanism
Source: Nat Struct Mol Biol. 2021 Sep 23;28(10):799–810. doi: 10.1038/s41594-021-00656-9 (PMC8500839; doi:10.1038/s41594-021-00656-9)
Supplement: Supplementary file 2 — Reporting Summary [file 41594_2021_656_MOESM2_ESM.pdf]

nature research

Corresponding author(s): Kai Zhang

Last updated by author(s): Jul 21, 2021

Reporting Summary

Nature Research wishes to improve the reproducibility of the work that we publish. This form provides structure for consistency and transparency in reporting. For further information on Nature Research policies, see our Editorial Policies and the Editorial Policy Checklist.

Statistics

For all statistical analyses, confirm that the following items are present in the figure legend, table legend, main text, or Methods section.

n/a	Confirmed	
☐	☒	The exact sample size (*n*) for each experimental group/condition, given as a discrete number and unit of measurement
☐	☒	A statement on whether measurements were taken from distinct samples or whether the same sample was measured repeatedly
☐	☒	The statistical test(s) used AND whether they are one- or two-sided<br>*Only common tests should be described solely by name; describe more complex techniques in the Methods section.*
☒	☐	A description of all covariates tested
☒	☐	A description of any assumptions or corrections, such as tests of normality and adjustment for multiple comparisons
☐	☒	A full description of the statistical parameters including central tendency (e.g. means) or other basic estimates (e.g. regression coefficient) AND variation (e.g. standard deviation) or associated estimates of uncertainty (e.g. confidence intervals)
☐	☒	For null hypothesis testing, the test statistic (e.g. *F*, *t*, *r*) with confidence intervals, effect sizes, degrees of freedom and *P* value noted<br>*Give P values as exact values whenever suitable.*
☒	☐	For Bayesian analysis, information on the choice of priors and Markov chain Monte Carlo settings
☒	☐	For hierarchical and complex designs, identification of the appropriate level for tests and full reporting of outcomes
☒	☐	Estimates of effect sizes (e.g. Cohen's *d*, Pearson's *r*), indicating how they were calculated

Our web collection on statistics for biologists contains articles on many of the points above.

Software and code

Policy information about availability of computer code

Data collection	SerialEM 3.7.8
Data analysis	Relion 3.0, MotionCor 2, Gctf 1.08, Gautomatch 0.56, Cryosparc 2.12, Coot 0.8.9, Molprobity 4.5, PyMOL 2.3.2, Chimera 1.12, ChimeraX 0.6, Fiji, Refmac 5.7, IMOD 4.9.12, FIESTA 1.6.0

For manuscripts utilizing custom algorithms or software that are central to the research but not yet described in published literature, software must be made available to editors and reviewers. We strongly encourage code deposition in a community repository (e.g. GitHub). See the Nature Research guidelines for submitting code & software for further information.

Data

Policy information about availability of data

All manuscripts must include a data availability statement. This statement should provide the following information, where applicable:

- Accession codes, unique identifiers, or web links for publicly available datasets
- A list of figures that have associated raw data
- A description of any restrictions on data availability

The coordinates are deposited in the Protein Data Bank with PDB IDs 7K58 (OAD-MTD in MTBS-1), 7K5B (OAD-MTD in MTBS-2), 7KEK (free OAD in pre-parallel conformation), 7N32 (four PFs of OAD-MTD), 7MWG (16-nm MTD), respectively. The cryo-EM maps are deposited in the Electron Microscopy Data Bank with IDs EMD-22677 (OAD-MTD in MTBS-1), EMD-22679 (OAD-MTD in MTBS-2), EMD-22840 (free OAD in pre-parallel conformation), EMD-24066 (16-nm MTD). Source data are provided with this paper as well.

April 2020

Field-specific reporting

Please select the one below that is the best fit for your research. If you are not sure, read the appropriate sections before making your selection.

☒ Life sciences ☐ Behavioural & social sciences ☐ Ecological, evolutionary & environmental sciences

For a reference copy of the document with all sections, see nature.com/documents/nr-reporting-summary-flat.pdf

Life sciences study design

All studies must disclose on these points even when the disclosure is negative.

Sample size	More than 50 microtubules were randomly selected for velocity analysis in the gliding assay. More than 15,000 cryo-EM movies were collected to achieve near-atomic reconstructions. 268, 712 high-quality particles were included in the final reconstruction. For the inter-PF angle calculation, all reconstructed tubulins were taken into consideration.
Data exclusions	Electron microscopy: All micrographs were checked manually, and bad ones were excluded.<br>Microtubule gliding assay: Tracking results were manually inspected to exclude immobile filaments, surface dirt particles, tracks less than 1 second, and tracking errors due to filament collisions.<br>Other: No data was excluded from the analysis.
Replication	OAD purifications were repeated over 5 times. The microtubule gliding assays were repeated three times using more than 50 microtubules to estimate the velocities. The OAD array reconstitution assays were repeated for more than 5 times. Cryo-EM structures were lowpass filtered and re-refined, which generated consistent reconstructions.
Randomization	Microtubules were randomly selected from different areas of microscopy slides without preference to estimate the gliding velocity.
Blinding	Blinding was not relevant to this study. We cannot be blinded as microtubules and OAD molecules have to be identified based on experience and prior knowledge of the field.

Reporting for specific materials, systems and methods

We require information from authors about some types of materials, experimental systems and methods used in many studies. Here, indicate whether each material, system or method listed is relevant to your study. If you are not sure if a list item applies to your research, read the appropriate section before selecting a response.

Materials & experimental systems

n/a	Involved in the study	
☒	☐	Antibodies
☐	☒	Eukaryotic cell lines
☒	☐	Palaeontology and archaeology
☒	☐	Animals and other organisms
☒	☐	Human research participants
☒	☐	Clinical data
☒	☐	Dual use research of concern

Methods

n/a	Involved in the study	
☒	☐	ChIP-seq
☒	☐	Flow cytometry
☒	☐	MRI-based neuroimaging

Eukaryotic cell lines

Policy information about cell lines

Cell line source(s)	Tetrahymena Thermophila SB715
Authentication	None of cell lines were authenticated.
Mycoplasma contamination	Mycoplasma contaminaiton was not performed in this study.
Commonly misidentified lines<br>(See ICLAC register)	No commonly misidentified lines were used in our study.
