# Supplementary figures and images for: Structures of outer-arm dynein array on microtubule doublet reveal a motor coordination mechanism
Source: Nat Struct Mol Biol. 2021 Sep 23;28(10):799–810. doi: 10.1038/s41594-021-00656-9 (PMC8500839; doi:10.1038/s41594-021-00656-9)

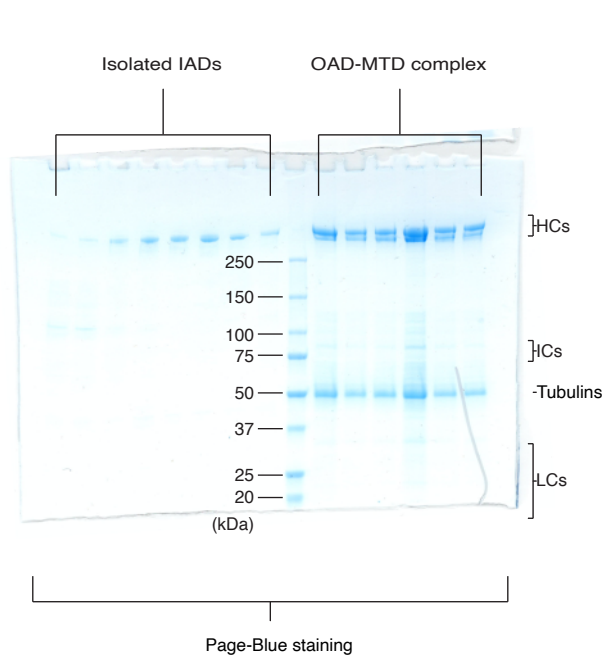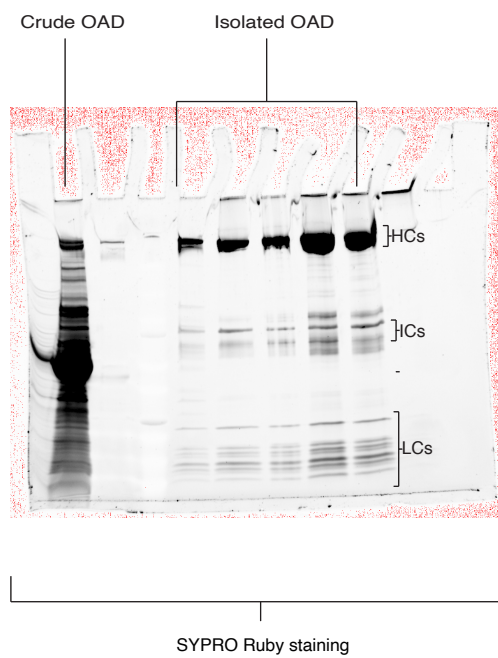

Supplement: Source Data Fig. 1 — Unprocessed gels. [file 41594_2021_656_MOESM7_ESM.pdf]
